# Assembling Disease Networks From Causal Interaction Resources

**DOI:** 10.3389/fgene.2021.694468

**Published:** 2021-06-11

**Authors:** Gianni Cesareni, Francesca Sacco, Livia Perfetto

**Affiliations:** ^1^Department of Biology, University of Rome Tor Vergata, Rome, Italy; ^2^Department of Biology, Fondazione Human Technopole, Milan, Italy

**Keywords:** network medicine, logic modeling, causality resources, prior knowledge network, causal interactions

## Abstract

The development of high-throughput high-content technologies and the increased ease in their application in clinical settings has raised the expectation of an important impact of these technologies on diagnosis and personalized therapy. Patient genomic and expression profiles yield lists of genes that are mutated or whose expression is modulated in specific disease conditions. The challenge remains of extracting from these lists functional information that may help to shed light on the mechanisms that are perturbed in the disease, thus setting a rational framework that may help clinical decisions. Network approaches are playing an increasing role in the organization and interpretation of patients' data. Biological networks are generated by connecting genes or gene products according to experimental evidence that demonstrates their interactions. Till recently most approaches have relied on networks based on physical interactions between proteins. Such networks miss an important piece of information as they lack details on the functional consequences of the interactions. Over the past few years, a number of resources have started collecting causal information of the type protein A activates/inactivates protein B, in a structured format. This information may be represented as signed directed graphs where physiological and pathological signaling can be conveniently inspected. In this review we will (i) present and compare these resources and discuss the different scope in comparison with pathway resources; (ii) compare resources that explicitly capture causality in terms of data content and proteome coverage (iii) review how causal-graphs can be used to extract disease-specific Boolean networks.

## Introduction

The term precision or personalized medicine reflects the motivation of using high content molecular information for disease diagnosis and for the design of effective personalized therapies (Ginsburg and Phillips, 2018). Advances in experimental methods, such as deep sequencing and high content proteomics (Nilsson et al., 2010; Goldman and Domschke, 2014), have enabled the comprehensive assessment of a patient's molecular profile in a time- and cost-effective manner. Patients' genomic and expression profiles are becoming increasingly more important diagnostic readouts and are likely to become soon compatible with clinical practice in most public hospitals. Whether patients can benefit from this promising treatment strategy on a large scale still remains uncertain (Zhang et al., 2020).

One main limitation of this genomic-approach is the lack of an effective strategy to extract clinically relevant information from these dense and noisy datasets. Network representation of biological complexity and graph theory are playing an increasingly important role in dealing with the intricacy of human physiology and pathology. Network-based approaches are used, in the context of a relatively new discipline dubbed “network medicine,” to address the interplay of the molecular mechanisms underlying complex diseases (Barabási et al., 2011). The main idea behind is that a network, where interactions between its components are represented in the form of a graph, provides a powerful mathematical framework for analysis and visualization of experimental results.

According to this vision, gene products govern cell physiology by interacting in a large interconnected network whose equilibrium is responsible for the dynamic homeostasis of “healthy” cells. The network properties are believed to be rather robust and resilient to perturbations of many of the nodes and some of their connecting edges. A few nodes of the network, however, are quite sensitive and their knock out or hyperactivation may cause large changes of the network properties leading to disease (Brinkman et al., 2006). Alternatively, and more frequently, a combination of alterations of the activities of nodes that, on their own, have little effect may synergize to alter the properties of sensitive regions of the network thereby leading to a pathological condition (Barabási et al., 2011). It is anticipated that the overlay of a patient genomic profile onto such a comprehensive cell network, or part of it, will provide a framework to help in patient diagnosis and therapy choice.

Cell networks are assembled from experimental evidence of physical or functional links between biological entities. This information is often difficult to retrieve and organize as it is dispersed in millions of scientific reports. In addition, experimental results are mainly reported in natural language that is not easily processed by computers. Thus, network approaches mostly rely on the work of database curators that, assisted by natural language processing tools, identify relevant reports in literature repositories and annotate the interaction evidence in a structured machine-readable format. Over the past few decades different players have engaged in the task of capturing evidence of protein interactions. Protein interaction is a generic term including different types of physical and functional relationships between proteins as identified by diverse experimental approaches (Zhou et al., 2016). Databases that aim at capturing this information have distinct focus and adopt models that best adapt to their scope. As a consequence, comparing and merging the data from the diverse databases is made difficult by the heterogeneity of the interaction types and the models to represent them.

A recent review by Touré et al. (2020) has discussed the different types of protein interaction resources focusing on a comparison of the adopted data structures and the data exchange and conversion procedures. Here we go over the models that have been adopted to represent experimental evidence of protein relationships mediating physiological and pathological processes. More specifically, we confront physical and causal interactions by briefly describing their characteristics and the resources that aim at capturing and organizing the two different interaction types.

**Figure 1:**
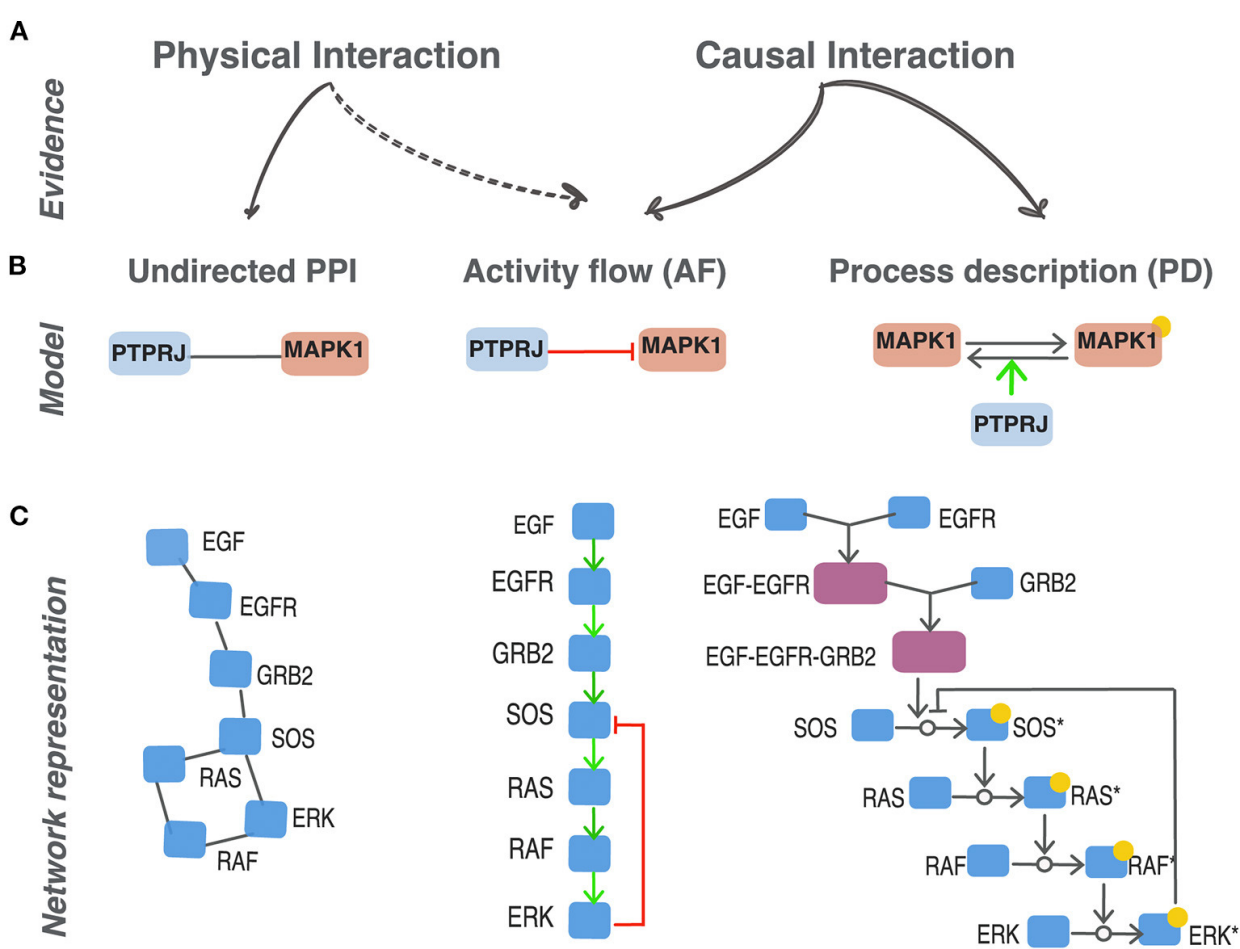
Different representations of protein interactions. **(A)** Experimental methods can either provide evidence that support a physical contact between two proteins to form a complex (physical interaction) or a modulation of the activity of a target protein caused by the activity of a parent protein (causal interaction). **(B)** Different graphical representation of the same biological statement: PTPRJ dephosphorylates and inhibits MAPK1 (Sacco et al., 2009). Three distinct models to represent protein relationships supported by different experimental evidence: undirected PPI, activity-flow and process description. **(C)** The EGFR signaling pathway represented as an undirected protein-protein interaction network (PPI), as activity-flow network (AF) and a process-description network (PD). *indicates the modified form of a given protein node.

We focus on resources that annotate causal interactions modeled as “activity-flow” (AF) networks (Figure 1) by considering and comparing their coverage and merits in different use cases. We will also present tools and strategies that make use of networks assembled from prior knowledge (PKNs) to produce executable logic models replicating phenotypes of clinical relevance. Finally, we discuss whether the evidence on causal relationships that is presently reported in the scientific literature is adequate to assemble a cell network of sufficiently high coverage and accuracy to be of clinical relevance.

## Resources Capturing Signaling Interactions

### Physical and Causal Interactions

Proteins interact in the cell forming a complex ordered functional mesh. Some of these interactions are necessary for maintaining cell organization whereas others support the cell response to internal and external stimuli, and are often transient (Acuner Ozbabacan et al., 2011). A variety of approaches suitable for high throughput analysis have been used to reveal the physical contacts between proteins without informing on the dynamic of signal propagation (Xing et al., 2016). More than 400 K “physical interactions” between human proteins have been reported in the literature by using these methods and for 85% of the proteins in the human proteome we know at least one physical partner in public databases (Orchard et al., 2014; Oughtred et al., 2021). Physical interactions are symmetrical by nature and, having no directionality, are represented as “undirected” graphs (Figure 1A). Transient signaling interactions, on the other hand, are often short lived and as such may not be revealed by the methods developed for physical interactions. They are often causal as one of the partners, the regulator, causes a functionally relevant modification of the target protein. These latter types of interactions may be modeled in two ways that are often referred to as “process descriptions” (PD) and “activity-flow” (AF) (Figure 1) (Le Novère, 2015; Türei et al., 2016; Touré et al., 2020).

Let us consider, as an example, the experimental observation that the phosphatase *PTPRJ* (DEP1) binds to *MAPK1* (ERK2) and inactivates it by removing a phosphate (Sacco et al., 2009). As shown in Figure 1B, an “undirected PPI” model represents this statement as a link between *PTPRJ* and *MAPK1* that has no direction. The “activity-flow” (AF) model, on the other hand, renders this information as a binary interaction where the two proteins are connected by an edge that has direction from *PTPRJ* to *MAPK1* and a sign which is graphically symbolized with a specific edge-form or color. This representation captures the evidence that *PTPRJ* is the regulator and *MAPK1* the target and that this interaction has the consequence of inactivating *MAPK1*. AF models offer the advantage of being represented as a set of binary interactions in a signed directed graph which is more informative than an undirected graph. Finally, the PD model captures additional mechanistic details. In this representation the target entity, *MAPK1* in our example, is split into two nodes representing the phosphorylated and unphosphorylated forms of the protein. The two forms are connected by a directed edge symbolizing the transition from one form to the other. The activity of the regulatory protein *PTPRJ* is represented as an edge promoting the removal of the phosphate from *MAPK1*. A limitation of this latter model is that the impact of the phosphorylation on the activation status of *MAPK1* cannot be directly derived; it is only implicit as it can only be inferred from the reconstruction of the downstream chains of reactions.

The different representations serve different purposes and answer different questions. For instance, analysis of highly connected regions of an undirected protein interaction network may reveal the formation of macromolecular complexes (Wang et al., 2009; Havugimana et al., 2012). Similarly, the function of a protein that is trapped in a subnetwork formed by proteins that are annotated to a specific biological process may provide hints on its function (Oliver, 2000). On the other hand, process description and activity-flow networks are appropriate to sketch the information flow from a receptor sensing a stimulus to activation of a transcription factor driving phenotype modulation.

Another major difference between physical and causal interaction datasets is proteome coverage as the latter have significantly lower coverage. This is partly due to incomplete curation of reported experimental evidence and partly to the lack of appropriate high throughput experimental approaches to reveal causal interactions on a large scale. In addition, many resources annotating PPI have, in recent years, joined their efforts forming a consortium (Orchard et al., 2014; Porras et al., 2020) for distributing curation investment and using common standards and curation rules, whereas causal resources have not reached such an agreement yet. For these reasons, many of the network approaches presently rely on networks based on physical protein interactions (PPI) (Zhang and Itan, 2019).

Approaches based on networks assembled by using information on causal relationships, however, are gaining momentum as they provide information that can be relatively easily converted into Boolean or ordinary differential equation models thus enabling users to compute the behavior of a system in different conditions (Le Novère, 2015).

Although there is no strict separation between the experimental evidence that can be captured by the different models, it is crucial to understand the data structure adopted by each resource as analyses built on information extracted from distinct databases may lead to different biological conclusions (Mubeen et al., 2019).

### Pathway Databases and Interaction Databases

Cell physiology is governed by a large connected network of physical and causal interactions. Nevertheless, biologists sometimes prefer to consider the cell model as an ensemble of unconnected pathways that, in a first approximation, function in isolation and do not crosstalk. However, this approximation neglects the effects of the cell network as a whole that may significantly affect the behavior of the pathway subnetworks. Although networks are useful abstractions, their functional integration into a cell model remains an important challenge. Capturing the experimental information for the assembly of protein interaction networks from primary literature data is an intimidating task. To assist scientists, over the past 20 years, a number of resources have set out to annotate an excerpt of the experimental facts related to protein interactions in structured formats in public repositories. However, different databases have been developed to serve different purposes, they adopt different curation policies and describe the same biological fact at different levels of abstraction and granularity.

**Figure 2:**
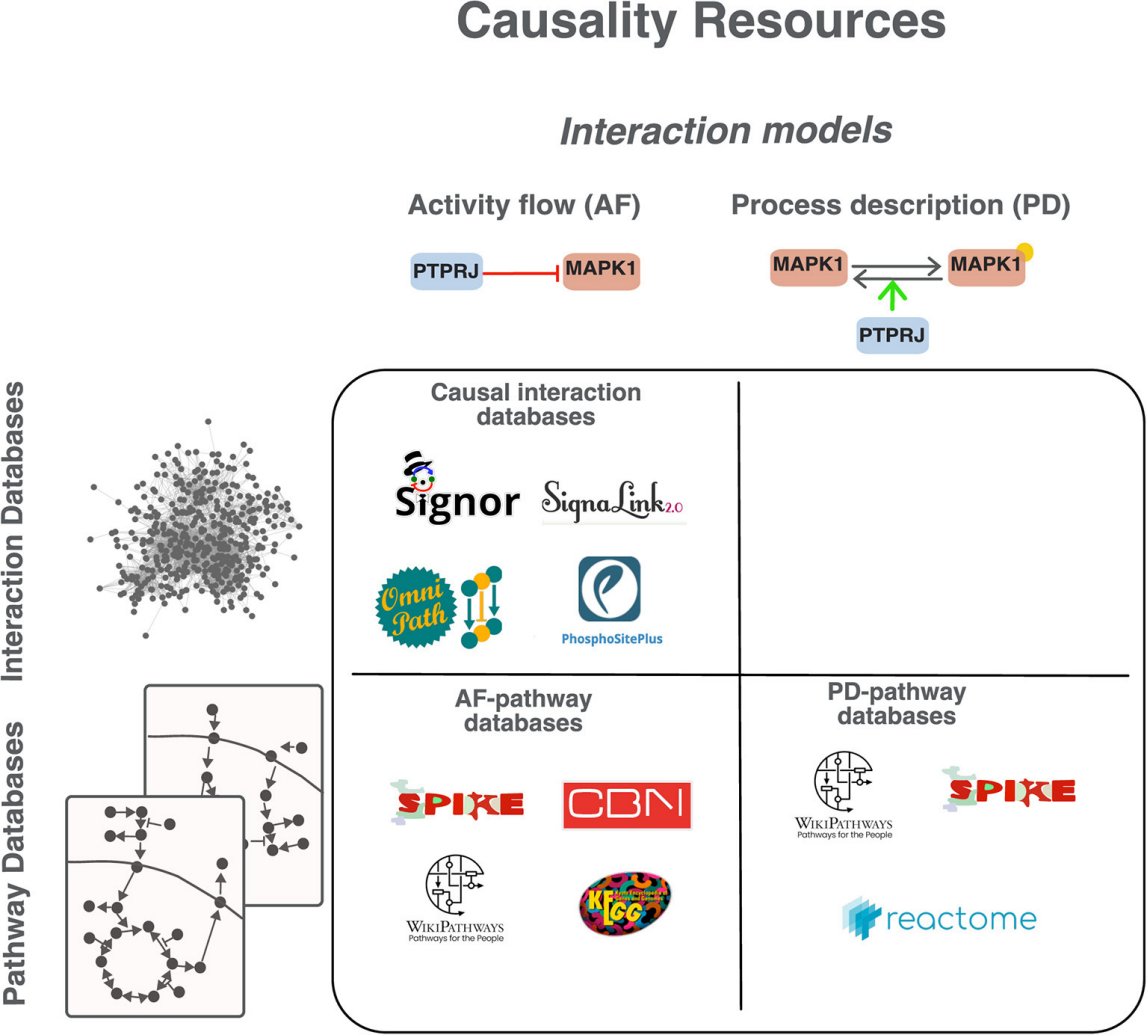
Classification of causality resources. Resources can be grouped according to the model adopted to represent causality in AF or PD (see Figure 1) and according to the organization of the information in “interaction databases” or “pathway databases.” In interaction databases relationships are annotated separately and not necessarily in the context of higher-level organizational structures, such as pathways. In “pathway databases” interactions are exclusively shown in the context of the pathway they participate in.

We here focus on resources that capture causality, hereafter referred to as “causality resources” (Figure 2). Considering the chosen representation model, databases can be grouped into two broad classes (Figure 2, Supplementary Table 1): “interaction databases,” where relationships are integrated in a global network and “pathway databases,” where interactions are curated and displayed in the context of the pathway they participate in.

The three most popular pathway resources are KEGG, Reactome and WikiPathways (Kanehisa and Goto, 2000; Slenter et al., 2018; Jassal et al., 2020). Among the pathway databases those adopting AF as interaction model are KEGG, SPIKE (Paz et al., 2011) and CBN (Boué et al., 2015) (Supplementary Table 1). The signaling information annotated in these databases is reviewed by domain experts and covers more than 50% of the human proteome. Aside from their descriptive value in the representation of cell physiology, they have proven useful in the analysis and interpretation of -omics data when coupled with algorithmic approaches such as gene set enrichment analysis GSEA and signaling pathway impact analysis (SPIA) (Subramanian et al., 2005; Tarca et al., 2009; Sprent, 2011). However, they do not provide an integrated picture of cell functioning as interactions are accessible only in the context of pathways and miss to offer a holistic view. Another class of resources, including Cell Collective (Helikar et al., 2012), Biomodels (Malik-Sheriff et al., 2020), the GINsim repository (Naldi et al., 2009) and the PyBoolNet repository (Klarner et al., 2017) collect assembled logical models. Briefly, these resources store models curated and tested by different groups for specific projects. Users can download the models and adapt them to different purposes. However, the models do not necessarily follow a common annotation standard. As a consequence, the integration into larger models is often not straightforward.

A third class of resources, such as SIGNOR (Licata et al., 2020), SignaLink (Csabai et al., 2018), OmniPath (Türei et al., 2016; Ceccarelli et al., 2020) or PhosphoSitePlus (Hornbeck et al., 2019) annotate interactions without necessarily listing them as members of a pathway. We will refer to these with the generic term “causal interaction databases” (Figure 2). This organization of the interaction data, which is not pathway centric, allows users to assemble an integrated cell network where all pathways are connected, thereby allowing to monitor pathway crosstalk.

## Activity-Flow Resources Comparison

With our contribution we intend to show how AF interactions from the different resources can be used to build logic networks to support modeling studies. To this end, we compare four major AF resources, KEGG, PhosphoSitePlus, SignaLink and SIGNOR.

These resources were selected as they are open-source, established, and popular as evinced from citation counts. In addition, they exclusively adopt “activity-flow” as a representation model (Supplementary Table 1). These databases are, however, highly heterogeneous in scope, and do not follow a common standard for the annotation and the export of the data (Dräger and Palsson, 2014). To address this issue the proteomic standard initiative for molecular interaction (PSI-MI) (Orchard, 2014) and the Gene Regulation Ensemble Effort for the Knowledge Commons (GREEKC) (https://www.greekc.org/) communities have recently developed CausalTAB, a common standard for exchange of causal information (Perfetto et al., 2019). However, of the four databases considered here, only SIGNOR presently offers to download its curated dataset in this format. As a consequence, the organization of the datasets for the comparison reported here turned out to be a substantial effort (Supplementary File 1 in Supplementary Material). To facilitate the task of integrating the information that can be downloaded from the different resources, OmniPath has embarked on a project aimed at merging the causal information from a large number of primary resources. This resource was also included in our analysis.

We designed this comparison to help non-computational scientists to incorporate computational modeling into their experimental practice. We point out that the comparison is limited to the portion of AF interactions that satisfy specific criteria and that some datasets (e.g., KEGG) might represent a subset of the total number of interactions that are annotated in the database.

The four primary resources considered here have a different focus and include different entity types as nodes in the network. For instance, KEGG and SIGNOR also annotate complexes. In addition, SIGNOR considers a wider range of entities including “phenotypes,” “stimuli” and “chemicals.” SignaLink and SIGNOR also curate indirect interactions. To harmonize the data in order to attain a fair comparison, we filtered the datasets to retain only direct causal interactions between human protein pairs (Supplementary File 1 in Supplementary Material). In addition, we only considered those relationships that are annotated with a literature reference. In this first comparison two entries are considered coincident if they involve the same protein pair with matching directionality, irrespective of the effect (activation/inhibition) of the interaction.

**Figure 3:**
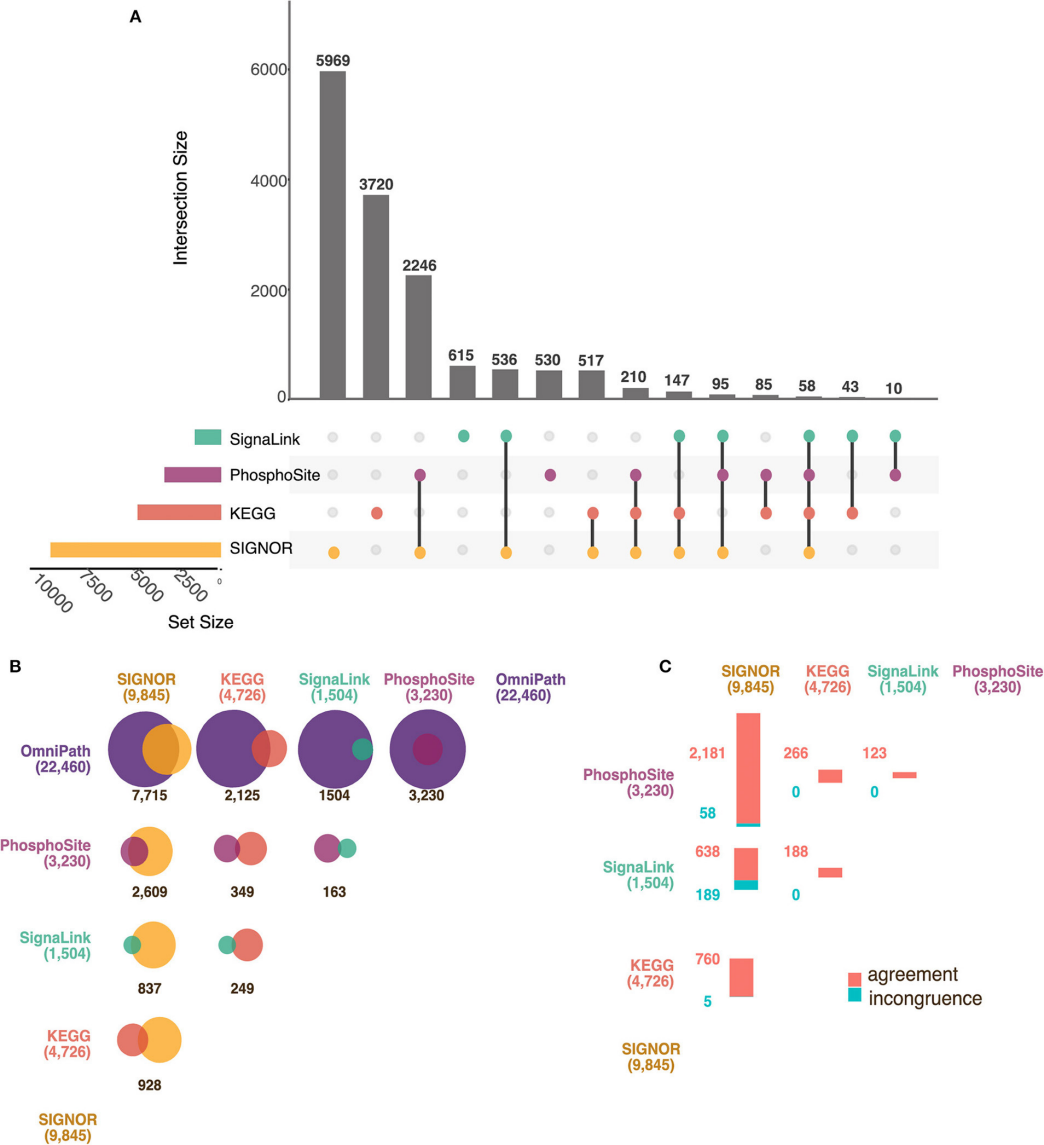
Comparison of AF Databases. **(A)** UpSet Plot showing the overlaps between four primary AF resources: SIGNOR (in yellow), KEGG (in red), PhosphoSitePlus (in purple) and SignaLink (in green). The vertical bars show the number of intersecting protein pairs (regulator-target) between resources, identified as connected colored circles below the histogram. The length of the horizontal bars is proportional to the dataset size of each resource. As an example, PhosphoSitePlus, SIGNOR and KEGG share 210 interactions. **(B)** Proportional Venn diagrams showing the overlap between the datasets of the four primary AF resources and OmniPath: SIGNOR (in yellow), KEGG (in red), PhosphoSitePlus (in purple), SignaLink (in green) and OmniPath (dark purple). Individual set sizes are in parenthesis. **(C)** Matrix of bar plots showing the number of interactions between pairs of proteins whose effect, up- down-regulation is annotated in an opposite way in each pair of primary resources. Agreement and disagreement are shown in red and blue, respectively.

In Figure 3A we show in an UpSet plot (Lex et al., 2014) the number of causal relationships that are annotated only in each of the databases or are common to all dataset combinations. We first notice that the four primary databases are largely complementary as more than 70% of the information is captured by only one database while fewer than 4% of the interactions (510) are annotated in three or four resources. SIGNOR with its 9,845 entries is the primary database with the highest number of entries. Still 5,003 entries of the remaining three primary resources are not in SIGNOR (Figure 3A). This complementarity of the datasets has motivated the OmniPath team to integrate all the causal information in a single dataset.

In Figure 3B we have reported the results of the comparison as Venn diagrams. Each resource is represented as a circle of different color whose size is proportional to data content. The circles overlap for an area that is proportional to the number of interactions that are present in both databases. The largest overlap between primary databases is observed in the comparison between SIGNOR and PhosphoSitePlus as both resources have put investment in the coverage of phosphorylation reactions. As PhosphoSitePlus does not curate other types of causal relationships its overlap with the other resources is negligible.

OmniPath, which integrates information from more than 100 different primary databases, is by far the most inclusive resource. However, although OmniPath claims full integration of interaction data, only 39% of the KEGG dataset is included in OmniPath (Figure 3B). This is because the standard OmniPath dataset only takes into consideration referenced protein relationships, whereas a large fraction of KEGG interactions is not linked to the manuscripts providing the supporting experimental evidence. Other inconsistencies are the consequence of an infrequent synchronization of the OmniPath dataset with the release of the primary resources. Of note over 20% of the interactions in SIGNOR are not present in OmniPath (Figure 3B).

By adding to the OmniPath dataset the missing data from the four primary resources it is possible to assemble a network of causal interactions linking nearly 5,800 proteins (28% of the proteome) connected by 27,040 edges (Supplementary Table 2). Eighty four percent of these are only curated in one or two resources, while the remaining 16% in three or more.

### Consistency of Data Curation in the Different Resources

The conclusion of the analysis in the previous section is that, in order to increase coverage, users should consider collating datasets from different resources. However, in large curation efforts, in some instances, the same experimental evidence can lead different curators to different interpretations. In addition, experimental reports addressing the same biological question reach sometimes contrasting conclusions. Thus, it is not surprising to observe that a causal relation between protein A and protein B is annotated as activating by one database and inactivating by another. However, this represents a problem in the assembly of AF networks from an integrated dataset. To investigate how serious this issue was, we next assessed the fraction of causal relationships that are inconsistently annotated.

Ninety five percent of the edges that are curated by more than one database are consistently associated with either up- or down- regulation (Supplementary Table 2). About 3,200 interactions are annotated with the same consensus effect in at least three resources, thereby accounting for a high-confidence subset of causal interactions. Conversely, 5% of the pairs are associated with both up- and down- regulation in different databases. Besides trivial curation errors, some discrepancies might reflect differences in the annotation policies of the different primary resources. Alternatively, it could be the consequence of conflicting literature reports or complex effects of an interaction leading to clashing consequences on the target protein function. For instance, *GSK3*-mediated *MAF* phosphorylation leads both to transcriptional activation and to degradation of the target (Rocques et al., 2007).

To quantify this lack of consistency, we compared the datasets from the four primary repositories. For this analysis, we first filtered out from each dataset those pairs that in each database are annotated with both a positive and a negative effect as in the *GSK3-MAF* example mentioned earlier (internal “inconsistencies”). As shown in Figure 3C, the percentage of incongruent pairs between DBs is relatively small, SignaLink and SIGNOR are the two repositories showing the highest number (and percentage) of contradicting interaction annotation. This subset of conflicting pairs has already been discussed (Perfetto et al., 2016) and can be explained by the differences in annotation granularity adopted by the two resources. For instance, SIGNOR annotates the mechanisms (such as ubiquitination, phosphorylation, etc.) involved in the interaction and, when provided, also the modified residues, while SignaLink only provides information about the causal effect.

## Disease Networks

### Assembly of Large Disease Networks

**Figure 4:**
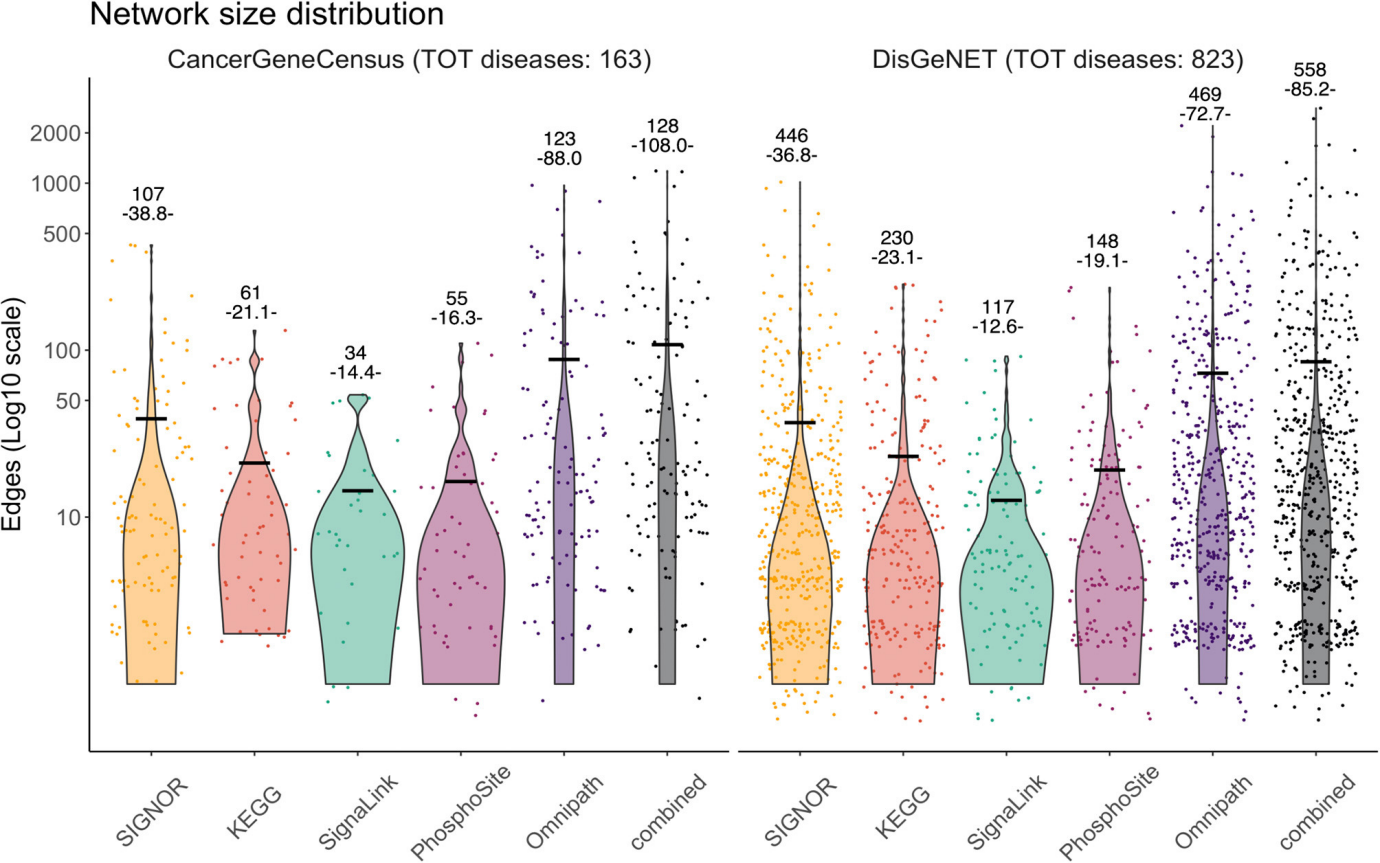
Violin plots illustrating the size distribution of the disease-networks that can be assembled by linking disease genes via causal interactions annotated in AF Databases. Disease-networks were assembled by using gene-disease associations (GDAs) downloaded from the Cancer Gene Census (left panel) (Sondka et al., 2018) and from DisGeNET selecting GDAs with score > 0.5 (right panel) (Piñero et al., 2020). The disease-networks also include proteins that directly connect disease gene products (bridge proteins) (Lo Surdo et al., 2018). Only diseases with at least two GDAs were considered in this analysis. Each dot represents a disease network and its size (y-axis) is defined as the number of edges that can be extracted from the five AF resources: SIGNOR (in yellow), KEGG (in red), PhosphoSitePlus (in purple), SignaLink (in green) and OmniPath (dark purple); and from a network derived by taking into considerations all the relationships annotated in at least one resource, combined (black). On top of each violin the total number of disease-networks that can be assembled by using the annotated causal relationships from each corresponding resource is displayed. In brackets we show the average size of the network, also indicated by a horizontal black bar.

We next asked whether the combined causal information captured by the different primary resources is sufficiently complete to be used to assemble informative disease networks linking most of the genes that are found mutated in patients. We used the expert curated information of the Cancer Gene Census (Sondka et al., 2018) that annotates 389 cancer types with lists of genes observed to be significantly mutated in cancers. Similarly, we used the information collected by the DisGeNET resource (Piñero et al., 2020) to download lists of gene-disease associations (GDAs) for 4,713 polygenic diseases (DisGeNET score > 0.5). These lists have different sizes ranging from one up to 83 genes in the case of “Malignant neoplasm of breast.” We filtered the lists by selecting diseases with at least two genes annotated, 163 and 823 diseases in Cancer Gene Census and DisGeNET, respectively (Figure 4). These disease-gene lists were used to query the AF resources for interactions linking the disease genes. We also included in the network the proteins that by forming a bridge between the query proteins, allow to connect them. The rationale for inclusion of “bridge proteins” is further discussed in the next paragraph.

The results of the approach are shown in Figure 4 as violin plots illustrating the distribution in the number of edges in the networks assembled by this automatic procedure. As proteome coverage is far from being complete, not all disease gene lists could be connected to form a network in the different resources. Above each violin we have indicated the number of diseases for which it was possible to assemble a network by interrogating each of the resources together with the average network size (average number of edges). As a larger coverage corresponds to a higher number of connections, retrieving interactions from SIGNOR allowed the assembly of a higher number of disease networks (446 and 107 from the DisGeNET and Cancer Gene Census lists, respectively) in comparison with the other primary resources. Similarly, SIGNOR-derived networks tend to be larger, in terms of number of connections. OmniPath that integrates all the primary databases allows an even higher coverage (both in terms of number of diseases and in average network size). However, as already noted, by integrating the data of the four primary resources and OmniPath an even higher number of disease genes could be assembled into connected networks.

It is finally to note that among the resources compared here, only SIGNOR and OmniPath have implemented a web tool to extract connections between a list of input proteins and to return the results either in graph or table format. To apply a similar procedure to the dataset offered by the other databases dataset-manipulation and/or parsing is necessary.

### The Gray Platelet Syndrome

**Figure 5:**
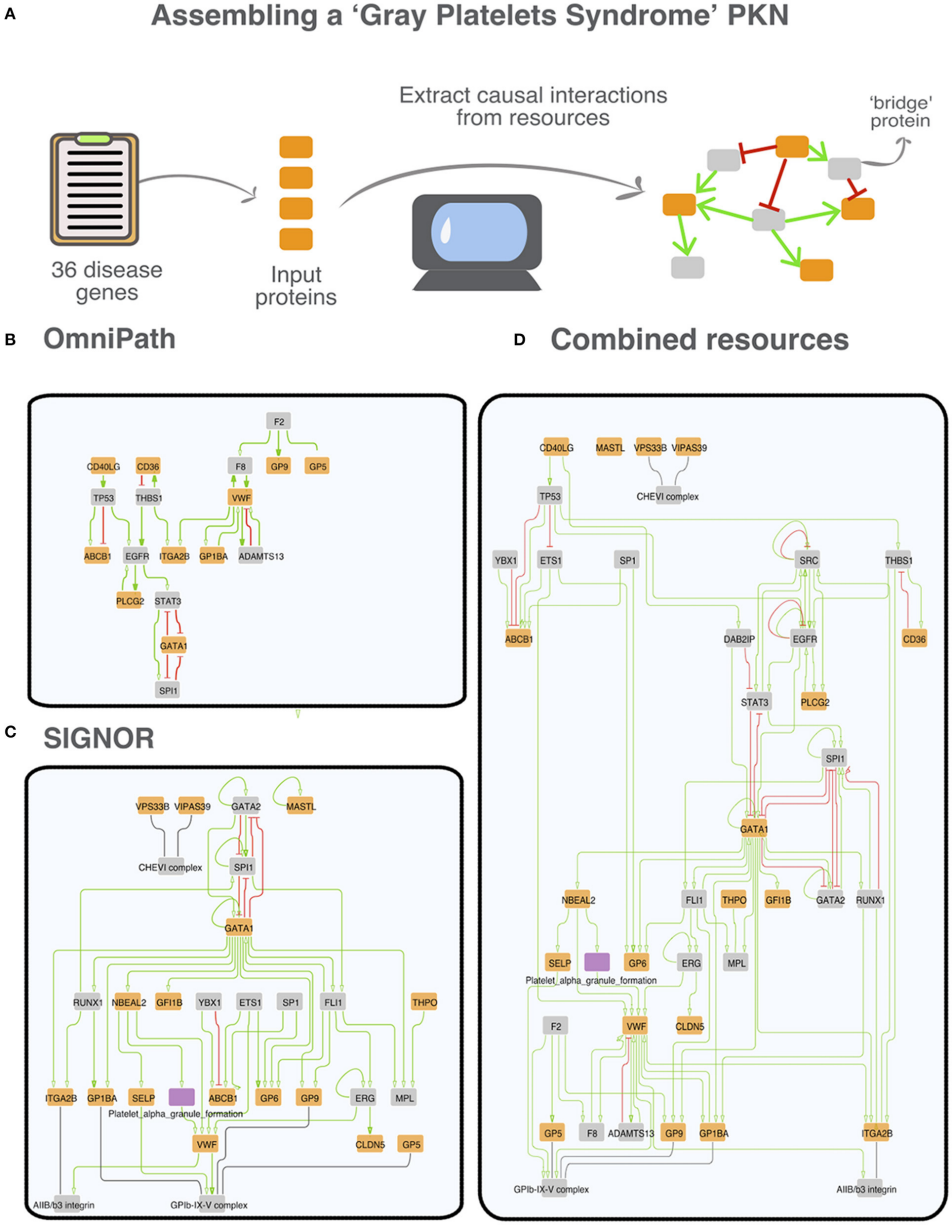
A prior knowledge network (PKN) associated with the “Gray Platelet syndrome.” **(A)** Strategy to derive the networks from the causal data in each resource. Thirty six gene-disease associations for the Gray Platelet syndrome were downloaded from MalaCards (Rappaport et al., 2017). Disease genes are used as seeds (orange nodes) to assemble the networks by searching causal resources for connecting relationships. To implement this strategy, we searched data from primary resources, from OmniPath; and from a virtual resource integrating all the datasets. Up - or down-regulations are illustrated in the graphs as green arrows and red t-shaped edges, respectively. We also included bridge proteins (gray nodes). Bridge proteins are proteins that connect at least two seed proteins (Lo Surdo et al., 2018). We were not able to obtain a significant network (>2 interactions) from KEGG, PhosphoSitePlus and SignaLink. **(B)** Network extracted from OmniPath: 18 nodes and 27 edges. **(C)** Network extracted from SIGNOR: 29 nodes and 53 edges. The purple node corresponds to the phenotype “platelet alpha granule formation,” annotated in SIGNOR (Licata et al., 2020). **(D)** Network that can be derived by combining the datasets annotated by the five combined resources: 41 nodes and 96 edges.

As an example of the results that one obtains by the procedure detailed in the previous section we will describe in more detail the networks retrieved in the case of the Gray Platelet syndrome (GPS). GPS is a rare recessive autoimmune disorder characterized by a variety of symptoms including the absence of platelet alpha-granules, bleeding disorders and bone marrow fibrosis (Gunay-Aygun et al., 2010). *NBEAL2* is the most frequently mutated gene in patients affected by this condition. However, due to the rarity of GPS, the molecular mechanisms underlying the disease are still poorly understood (Gunay-Aygun et al., 2011). We first assembled a list of 36 GPS associated genes and used this list to interrogate the different primary datasets and OmniPath (Figure 5A). As shown in Figure 5, in network assembly we also included “bridge proteins,” nodes that link two “disease proteins.” One advantage of using “bridge proteins” is that they allow for the expansion of the search space and for the retrieval of a graph connecting most of the disease proteins. By applying the aforementioned method, we succeeded in retrieving networks with a relevant (>2) number of interactions only by interrogating SIGNOR and OmniPath (Figures 5B,C).

By combining all the interactions, we obtain the most detailed graph incorporating 41 nodes and 91 edges and connecting 19 of the 36 input proteins (Figure 5D). As phenotypes are entities in the SIGNOR dataset, the integrated network also includes the “Platelet alpha granule formation” phenotype. Including phenotype entities improves the readability of the graph and strengthens the biological significance of the derived network.

GPS is a rare and poorly characterized disease. The advantage of this approach is that it compensates the lack of information annotated in the literature about pathways and perturbed molecular events.

To compare the results obtained in the case of GPS to that of a highly characterized disease, we applied a similar strategy to “Malignant neoplasm of breast.” This tumor type has the highest number of GDAs (83) in the DisGeNET resource. Not surprisingly, the retrieved networks are larger than the ones obtained for GPS, including 2,562, 533, 115, and 563 edges for SIGNOR, KEGG, SignaLink and PhosphoSitePlus respectively; 8,430 for OmniPath; and 11,513 for the five resources combined together. Such networks are extremely complex and difficult to interpret and might require stricter search parameters or filtering options that provide contextualization of the network (see next paragraphs).

These observations support the notion that there is no unique strategy to extract a diseases-PKN from AF repositories and the choice of a search method should be guided by quality and amount of information available for that specific pathology.

## Logic Models From Prior Knowledge Networks

AF networks provide mechanistic details on the information flow in a biological system in physiological and pathological conditions thereby allowing one to explore the functional consequences of modulating the activity of any specific node. However, they are of little practical value if one wants to identify the equilibrium states of a system in varying contextual conditions. Different approaches have been developed to obtain predictive models, including differential equation-based models, rule-based, Bayesian network inference and logic-based models. Despite their simplicity, logic-based models (Boolean) have gained attention as, differently from modeling approaches based on ordinary differential equations, they can be applied to relatively large biological networks (Morris et al., 2010; Wang et al., 2012). Boolean models provide a simple yet powerful qualitative approach to describe how a system responds to contextual changes. The problem of assembling and contextualizing predictive Boolean models from prior knowledge and/or experimental data has been discussed and is further reviewed in section Conclusions and Perspectives (Vinayagam et al., 2011; Wang et al., 2012; Lages et al., 2018; Aghamiri et al., 2020; Dugourd et al., 2021).

The information embodied in activity-flow networks can be relatively easily converted into Boolean rules, where biological entities are modeled as Boolean variables whose activities are characterized by a simple On/Off behavior and where multiple incoming regulatory signals are integrated by logic gates. This qualitative approach approximates the response of a system and permits to address simple–albeit relevant- questions related to the phenotype that are favored in specific initial conditions or to the impact of a loss or gain of function mutations on any clinically pertinent phenotype.

Selvaggio and colleagues defined a logic model of the epithelial-to-mesenchymal transition that enabled the identification of new potential paths connecting microenvironmental signals to cancer cell plasticity (Selvaggio et al., 2020). Logic-based models have also been used to understand the molecular mechanisms underlying complex diseases. As an example, the group of Saez-Rodriguez has recently developed an approach combining *ex-vivo* high-throughput screenings of colon cancer biopsies with logic-based models. Their approach enabled them to generate patient-specific predictive models of apoptosis that can be used to rationally design personalized therapies (Eduati et al., 2020). Logic-based models have also been applied to explore whether and how the genomic context affects the behavior of a patient specific system. Béal et al. integrated mutation data, copy number alterations, and expression data into a breast-cancer logical model for clinical stratification of patients (Béal et al., 2018). Palma et al. built a Boolean model of acute myeloid leukemia whose predictions, once combined with patients' genomic profiles, correlate with clinical parameters, including patient life expectancy (Palma et al., 2021). Complex physiological processes such as hematopoiesis or macrophage differentiation can also be described by logic-based models of the different cell populations along the differentiation process (Collombet et al., 2017; Palma et al., 2018). Interestingly, logic-based models have also been used to discover novel anti-cancer drug combinations that efficiently kill cancer cell lines (Flobak et al., 2015).

## Conclusions and Perspectives

Resources that organize in a structured computer-readable format causal information between gene/proteins assist in the assembly of networks linking disease genes by logical connections. These in turn can be converted into logic models to predict phenotype modulation in different genomic contexts and under drug treatment.

Here we have focused on network strategies that make use of prior knowledge derived from low throughput experiments as annotated in public databases. These methods are somewhat biased as they depend on curators' decisions. It should be mentioned that alternative approaches based on reverse engineering allow researchers to draw networks in an unbiased manner by using genome wide gene expression data to infer relationships between genes (Pe'er and Hacohen, 2011). By these strategies, if two genes are co-expressed they are inferred to be functionally correlated and are linked in a gene regulatory network. Reverse engineering approaches, however, relying mostly on genome-wide expression studies, provide information on gene regulatory networks but say little about signaling networks where protein modification and modulation of stability play an important role that cannot be inferred from transcriptomics.

Although strategies based on prior knowledge have already shown some success, as reviewed here, we would like to conclude this contribution by discussing the current limits of these approaches and by identifying the areas where investment should be directed in the near future.

### Incomplete Coverage

At the time of our survey only ~28% of the proteome is integrated into a global cell network by the information captured in AF repositories. This represents a severe limitation as for many disease-genes we do not have any clue about the functional consequences of modulating their activities. This can, to some extent, be addressed by increasing the curation effort and perhaps by establishing a collaborative consortium of resources similar to the IMEx consortium in the PPI domain (Porras et al., 2020). However, we also have to accept that for many proteins we have hardly any experimental evidence about their functions, let alone their causal connections with the activity of other proteins in the cell network.

### Editing Automatically Generated Models

The networks that are derived by the strategy that we have delineated here are highly connected and complex and as such sometimes difficult to understand and model. Some interactions that are not supported by thorough evidence and repeatability or are implausible can be removed after a detailed review of the model connections by a domain expert. However, the development of automatic pruning methods is also desirable. For instance, not all the causal edges are equally supported by experimental evidence. The SIGNOR resource assigns to each causal relationship a score that reflects its experimental support. This can be used to filter the models and delete the connections with little experimental support. However, causal relationships are likely to depend on biological context. Thus, the scoring system should be made context/tissue specific. The increasing availability of tissue specific proteomic and (single cell) transcriptomic data (Fagerberg et al., 2014; Uhlén et al., 2015; Fernandez et al., 2019) should make this possible in a reasonably near future. Computational optimization methods such as CellNetOpt (Terfve et al., 2012), PRUNET (Rodriguez et al., 2015) or MetaReg (Ulitsky et al., 2008) can be used to identify the causal connections that are important to adapt models to context by monitoring their ability to reproduce the response of different cell systems to perturbations.

### Logic Gates

As briefly discussed in this review, an AF network can be easily converted into simple Boolean models. This conversion process is set back by the observation that proteins in an AF network often receive multiple inputs from upstream proteins and these inputs govern the activity of a node as a function of the activity of the upstream nodes at each cycle of a simulation. To establish the logic functions determining node activity one needs information on how to combine these inputs. For instance, if both the kinase and the phosphatase modulating the phosphorylation state of a substrate site are active, will the substrate be phosphorylated or not? This information cannot be extracted easily from the limited available experimental evidence and approximate approaches are often used. For instance, an inhibitor win approach was often used with some success (Dorier et al., 2016; Palma et al., 2021). Alternatively, once a PKN model has been assembled the connections and the logic gates can be optimized from the ability of different models to reproduce results of perturbation experiments (Terfve et al., 2012). Developments of reasonably high-throughput experimental methods to address this limitation are highly needed.

These considerations underscore the present limits of the approach that we have discussed. Nevertheless, some initial successes in modeling clinically relevant phenotypes, as we have detailed in this review, and the delineation of a strategy to address the current limits provide confidence that cell/disease specific logic models should soon contribute to diagnosis and therapeutic decisions in clinical practice.

## Author Contributions

GC and LP: conceptualization and supervision. LP: formal analysis and visualization. GC, FS, and LP: writing—original draft preparation and review and editing. All authors have read and agreed to the published version of the manuscript.

## Conflict of Interest

The authors declare that the research was conducted in the absence of any commercial or financial relationships that could be construed as a potential conflict of interest.
