# Supplementary material for: Assembling Disease Networks From Causal Interaction Resources
Source: Front Genet. 2021 Jun 11;12:694468. doi: 10.3389/fgene.2021.694468 (PMC8226215; doi:10.3389/fgene.2021.694468)
Supplement: Supplementary file 1 [file Data_Sheet_1.pdf]

Supplementary Material

1 Supplementary Data

Here we describe the key features of each of the five considered resources and we report how we extracted and filtered the causal datasets used in our comparison.

1.1 KEGG

KEGG PATHWAY (Kanehisa and Goto, 2000) is a collection of manually drawn pathway maps representing experimental knowledge on metabolism and various other signaling events. Each pathway map is represented as a network of molecular interactions and reactions annotated according to two different models. Metabolic pathways are represented as "process description" networks (see Fig.1A) while the remaining pathways, which include signal-transduction and other cellular processes, are shown as "activity-flow" networks (Fig. 1A). Maps in KEGG are linked to the genes and gene products that participate in the process, which are indicated as KEGG-internal identifiers. *Ad hoc* conversion tools are, however, available at https://www.kegg.jp/kegg/tool/conv_id.html. Interactions in KEGG maps have causal effect, where activations are depicted as arrows and inhibitions as T-shaped edges. Additional data such as the mechanism that underlies the causal effect is encoded in the edges: "+p" indicates phosphorylation, "+m" methylation etc. Each map is linked to one or more supporting publications.

The complete KEGG dataset cannot be freely downloaded as bulk downloading needs a subscription and payment of fees. For this reason, to extract the full human KEGG interactome for the scope of the comparison, we used the OmniPath web service (https://omnipathdb.org). We stress, however, that the dataset retrieved via OmniPath might not represent the complete KEGG interactome.

We retrieved the activity-flow interactions using the queries:

https://omnipathdb.org/interactions?genesymbols=yes&fields=sources,references&datasets=omnipat h,pathwayextra&resources=KEGG&license=ignore&password=catnip

For the TF-target interactions:

https://omnipathdb.org/interactions?genesymbols=yes&fields=sources,references&datasets=tf_target, dorothea&resources=KEGG&license=ignore&password=catnip

Importantly, this allowed us to incorporate every interaction in KEGG, also those not supported by literature evidence. Finally, we only selected direct and causal interactions involving proteins.

1.2 SignaLink 2.0

SignaLink (Csabai et al., 2018) is a multi-layered resource where a core of manually annotated signaling interactions is connected to additional layers of regulatory interactions integrated from external databases. Interactions are organized in 7 cellular pathways. However, the dataset can be fully

downloaded at http://signalink.org/download in a tabular format and no programming skills are required. When downloading data from SignaLink there is a possibility to set parameters and options by choosing from the main menu in the download section. In addition, it is possible to customize the download to select data relative to a specific species, pathway or layer.

Every interaction in SignaLink is modelled as activity flow, it has a causal effect, but no additional information about the mechanism (e.g. phosphorylation, ubiquitination etc.) is provided. Proteins are mapped to UniProtACs. For the scope of the comparison, we extracted from the resource dataset human, manually curated, direct and directional interactions.

SignaLink 2.0 is licensed under the Creative Commons Attribution-NonCommercial-ShareAlike 3.0 Unported License.

1.3 PhosphoSitePlus®

PhosphoSitePlus® (Hornbeck et al., 2019) is a repository focused on the manual annotation of experimentally observed post-translational modifications (PTM) with focus on phosphorylations. It is curated and updated regularly, and data can be downloaded at https://www.phosphosite.org/staticDownloads after free registration. For a number of PTMs the enzyme catalyzing the modification and the consequences on the activity of the target protein are also captured. These interactions can be modelled with an activity-flow representation. However, to this end it is necessary to extract and combine two types of evidence:

- PTM sites (e.g. acetylation, phosphorylation sites, etc.), where it is possible to find enzyme-substrate interactions and the amino acid modified by the enzyme;

- Regulatory sites, where a subset of the PTM sites is linked to a regulatory effect on the target protein. The regulatory effect is described with a controlled vocabulary. The remapping of some of the terms (e.g. "protein stabilization", "enzymatic activity, inhibited") to a causal effect (up- or down-regulation) is relatively straightforward, while for other terms (e.g. "apoptosis, altered", "cytoskeletal reorganization") the mapping is ambiguous.

In summary, the extraction of causal information from PhosphoSitePlus it requires the remapping of the CV to causal effects and the parsing of the information from two different downloadable files.

To facilitate the procedure, we accessed the data using the OmniPath web service (https://omnipathdb.org).

Also in this case, we want to stress that the dataset retrieved via OmniPath might not represent the complete PhosphoSitePlus interactome.

We extracted activity-flow interactions using the queries:

https://omnipathdb.org/interactions/?genesymbols=1&fields=sources,references&databases=PhosphoSite

https://omnipathdb.org/interactions/?genesymbols=1&fields=sources,references&databases=PhosphoSite_KEA

https://omnipathdb.org/interactions/?genesymbols=1&fields=sources,references&databases=PhosphoSite_MIMP

https://omnipathdb.org/interactions/?genesymbols=1&fields=sources,references&databases=PhosphoSite_ProtMapper

https://omnipathdb.org/interactions/?genesymbols=1&fields=sources,references&databases=PhosphoSite_noref

Finally, we combined the data and filtered the list to display only causal interactions between human proteins. PhosphoSitePlus®, created by Cell Signaling Technology is licensed under a Creative Commons Attribution-NonCommercial-ShareAlike 3.0 Unported License.

1.4 SIGNOR

SIGNOR (Licata et al., 2020) is a repository that stores signaling information modelled as activity-flow, with details about the mechanism (e.g. binding, phosphorylation, transcriptional activation, etc.) causing the up/down-regulation of the target entity. Entities can be proteins (identified by UniProtACs), chemicals, protein complexes, phenotypes and stimuli among others. Additional metadata such as modified residues and cell lines associated with the observed relationship are also associated to the entries. SIGNOR is updated regularly and users can download the whole dataset including the recently annotated entries. Data is regularly curated and updated and is freely accessible at https://signor.uniroma2.it/downloads.php as a simple tab delimited file or as PSI-MI CausalTab format (Perfetto et al., 2019). We downloaded from the website the entire human dataset and we filtered out interactions having EFFECT = "unknown" or involving non-protein entities. SIGNOR is licensed under a Creative Commons Attribution-NonCommercial 4.0 International (CC BY-NC 4.0) license.

1.5 OmniPath

OmniPath (Türei et al., 2016) is a secondary resource that has been developed to integrate information from primary DBs, allowing for a more comprehensive overview of the signaling interaction landscape. OmniPath mainly incorporates causal interaction data (e.g. activity-flow, enzyme-substrate and miRNA-RNA) from more than 100 resources. Interactions are remapped to UniProtACs and modelled as “activity-flow” networks. The "OmniPath" dataset in OmniPath consists of a selection of human interactions that represents an arbitrary optimum between coverage and quality (e.g. only referenced interactions are included). OmniPath can be accessed via a web service (https://omnipathdb.org)), as a dedicated APP in Cytoscape (Ceccarelli et al., 2020), or via the OmniPath web service. We extracted activity-flow interactions using the query:

https://omnipathdb.org/interactions/?genesymbols=1

Finally, we selected the data to contain only direct and directional interactions with causal effect.

The data redistributed by the OmniPath web service doesn't have a license, it is therefore necessary to refer to the license of each original resource.

2 Supplementary Figures and Tables

Supplementary Table 1. Summary description of Pathway and Interaction resources that capture causality.

Supplementary Table 2. Regulator-target pairs extracted from the AF resources: SIGNOR, KEGG, SignaLink, PhosphoSitePlus and OmniPath. Causal effects associated to each pair as extracted from original resources are indicated in the corresponding column.

3 References

Ceccarelli, F., Turei, D., Gabor, A., and Saez-Rodriguez, J. (2020). Bringing data from curated pathway resources to Cytoscape with OmniPath. *Bioinforma. Oxf. Engl.* 36, 2632–2633. doi:10.1093/bioinformatics/btz968.

Csabai, L., Ölbei, M., Budd, A., Korcsmáros, T., and Fazekas, D. (2018). SignaLink: Multilayered Regulatory Networks. *Methods Mol. Biol. Clifton NJ* 1819, 53–73. doi:10.1007/978-1-4939-8618-7_3.

Hornbeck, P. V., Kornhauser, J. M., Latham, V., Murray, B., Nandhikonda, V., Nord, A., et al. (2019). 15 years of PhosphoSitePlus®: integrating post-translationally modified sites, disease variants and isoforms. *Nucleic Acids Res.* 47, D433–D441. doi:10.1093/nar/gky1159.

Kanehisa, M., and Goto, S. (2000). KEGG: kyoto encyclopedia of genes and genomes. *Nucleic Acids Res.* 28, 27–30. doi:10.1093/nar/28.1.27.

Licata, L., Lo Surdo, P., Iannuccelli, M., Palma, A., Micarelli, E., Perfetto, L., et al. (2020). SIGNOR 2.0, the SIGnaling Network Open Resource 2.0: 2019 update. *Nucleic Acids Res.* 48, D504–D510. doi:10.1093/nar/gkz949.

Perfetto, L., Acencio, M. L., Bradley, G., Cesareni, G., Del Toro, N., Fazekas, D., et al. (2019). CausalTAB: the PSI-MITAB 2.8 updated format for signalling data representation and dissemination. *Bioinforma. Oxf. Engl.* 35, 3779–3785. doi:10.1093/bioinformatics/btz132.

Türei, D., Korcsmáros, T., and Saez-Rodriguez, J. (2016). OmniPath: guidelines and gateway for literature-curated signaling pathway resources. *Nat. Methods* 13, 966–967. doi:10.1038/nmeth.4077.
